# Supplementary material for: Cortex-wide neural interfacing via transparent polymer skulls
Source: Nat Commun. 2019 Apr 2;10:1500. doi: 10.1038/s41467-019-09488-0 (PMC6445105; doi:10.1038/s41467-019-09488-0)
Supplement: Supplementary file 1 — Supplementary Information [file 41467_2019_9488_MOESM1_ESM.pdf]

SUPPLEMENTARY INFORMATION

Cortex-wide neural interfacing via transparent polymer skulls

Ghanbari et al.

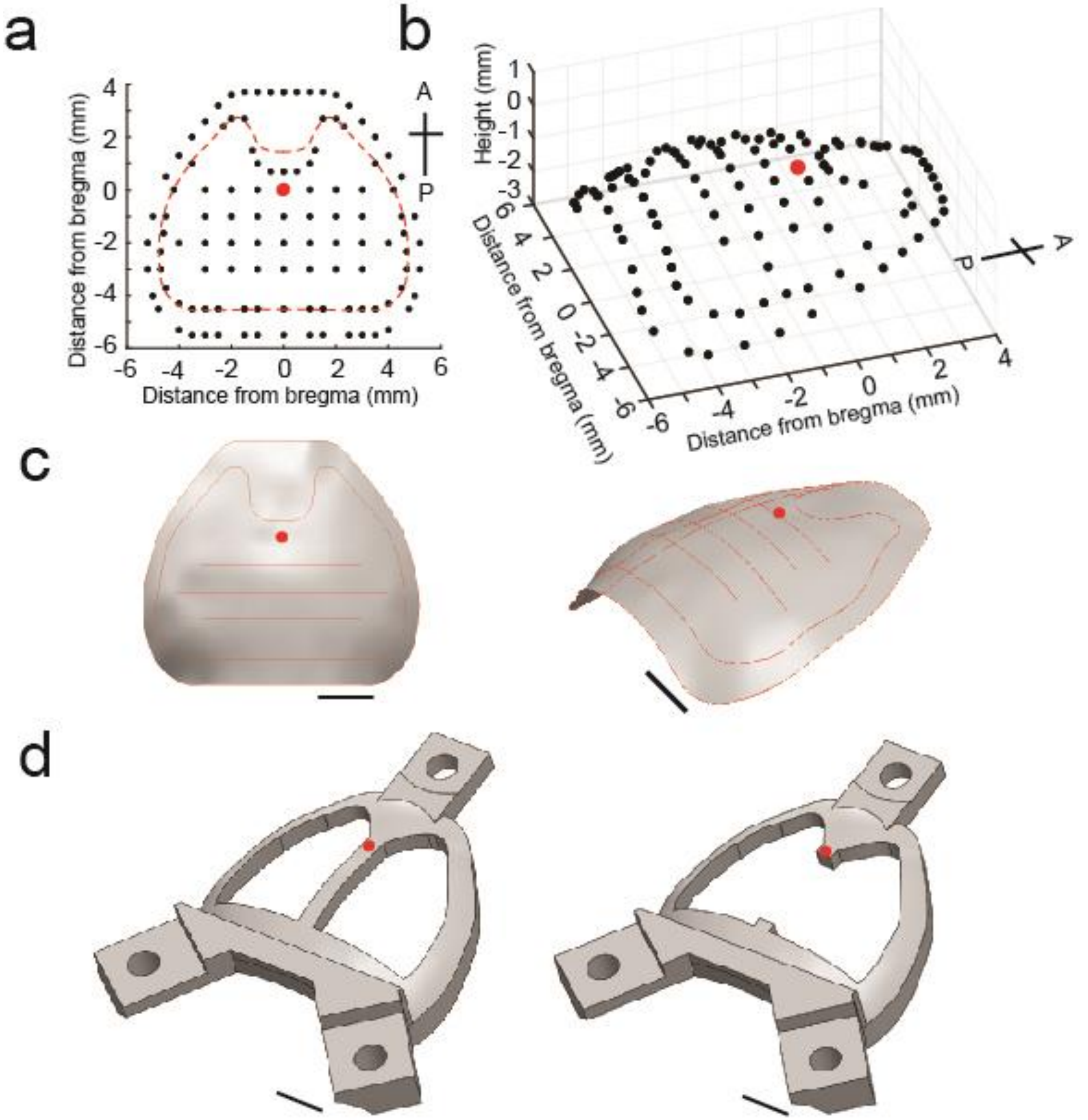

Supplementary Figure 1. See-Shells designed for C57BL/6 mice: (a) Lateral coordinates of 85 points profiled on the dorsal skull surface of a C57BL/6 mouse using the CNC mill in the stereotax (See **Methods**). The dashed line indicates predefined milling path used by the CNC mill during skull excision. **(b)** 3D point cloud generated from surface profiling at the points indicated in **(a)**. **(c)** 3D surface interpolated from the 3D point cloud shown in **(b). (d)** 3D CAD model of the See-Shell's PMMA frame. Two different versions were used in the experiments, with the version shown on the right used for 2P imaging. Red dots indicate the location of bregma. Scale bars in **c** and **d** indicate 2 mm.

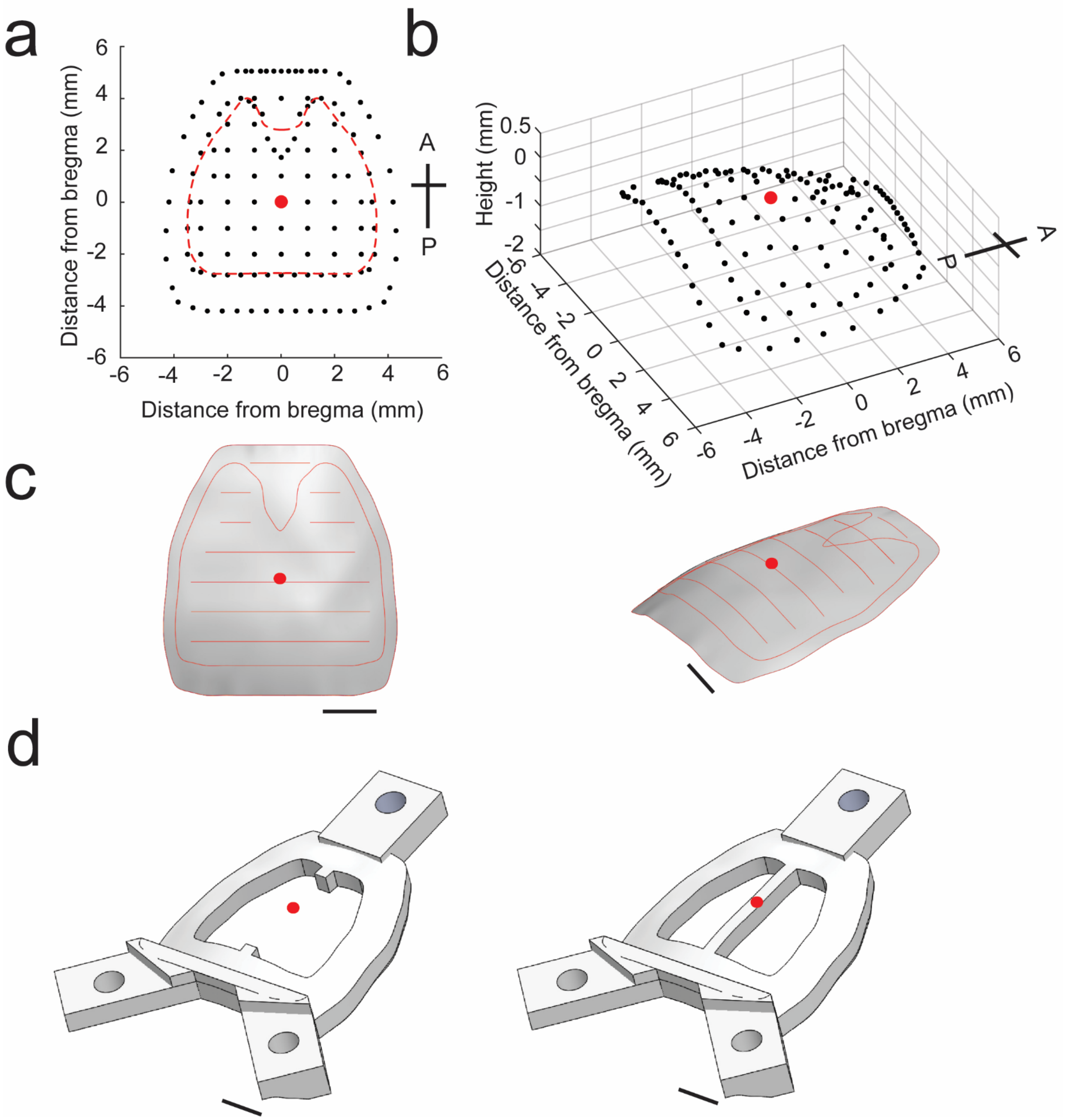

Supplementary Figure 2. See-Shells designed for *tg/tg* mice: (a) Lateral coordinates of 134 points profiled on the dorsal skull surface of a *tg/tg* mouse using the CNC mill in the stereotax (See **Methods**). The dashed line indicates predefined milling path used by the CNC mill during skull excision. **(b)** 3D point cloud generated from surface profiling at the points indicated in **(a)**. **(c)** 3D surface interpolated from the 3D point cloud shown in **(b)**. **(d)** 3D CAD model of the See-Shell's PMMA frame. Two different versions were used in the experiments, with the version shown on the right used for 2P imaging. Red dots indicate the location of bregma. Scale bars **c** and **d** indicate 2 mm.

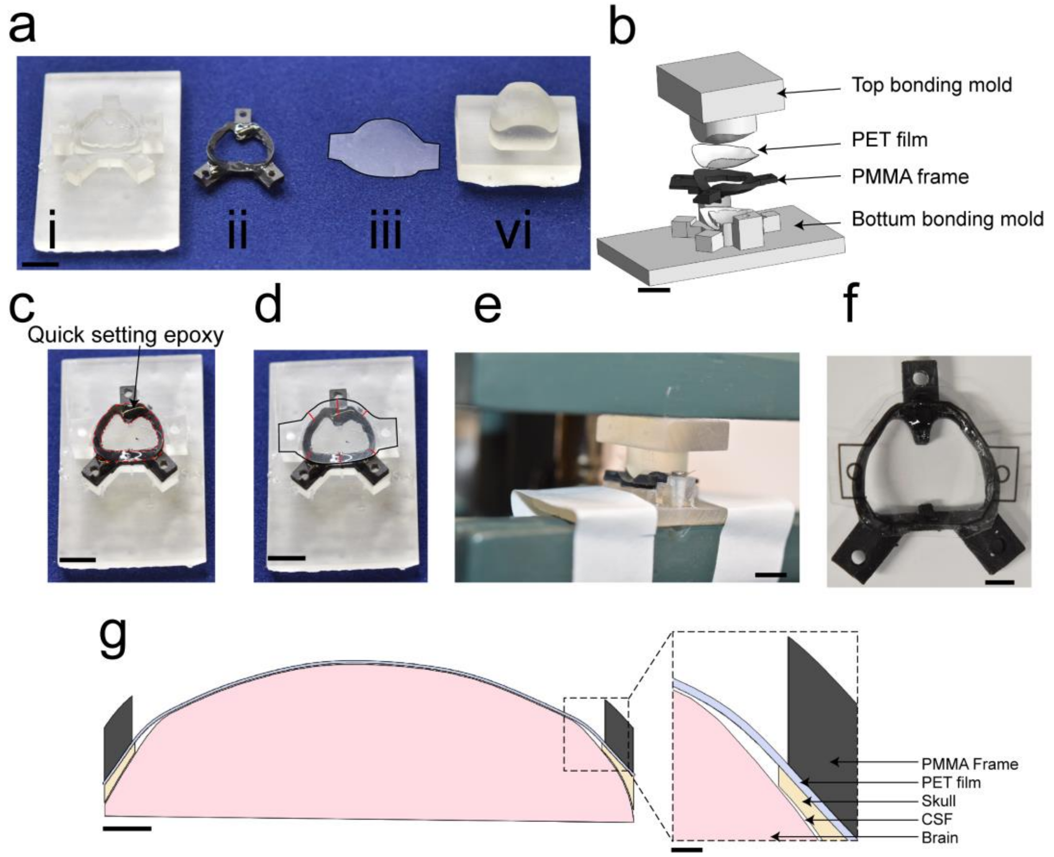

Supplementary Figure 3. See-Shell assembly and implantation: (a) (i) 3D-printed PMMA bottom bonding mold, designed based on the skull surface profile. (ii) 3D-printed PMMA frame of the See-Shell. (iii) PET film cut using a scissor around an outline roughly matching the PMMA frame. The outline is drawn using an erasable marker that is removed during the subsequent wash with ethanol. Extension on the sides help with handling the PET film with forceps during assembly. (vi) 3D-printed PMMA top bonding mold designed based on the skull surface profile. **(b)** CAD schematic of the 'sandwich structure' created when bonding the PET film to the frame. **(c)** The See Shell frame is retrieved from the 3D printer, washed with 100% isopropyl alcohol to remove uncured resin. The three holes are then tapped using a #0-80 tap. The bottom surface of the frame is coated with quick setting epoxy and mounted on the bottom mold**.** Dashed red borders indicate area of epoxy application. **(d)** PET film is aligned with the frame. Red lines indicate the location of relief cuts to assist with curving the PET film without strain. **(e)** PET is bonded to the PMMA frame by gently clamping it down using top and bottom bonding molds in a benchtop vise for 5-10 minutes. **(f)** The implant is removed from the clamp and the excess PET film surrounding the implant cut using a razor blade to realize final implant shown in **Figure 1**. **(g)** Cross-section cartoon of the entire implant on the skull illustrating how the PET sits on top of the exposed brain after craniotomy. CSF stands for cerebrospinal fluid. Scale bars in **a-e** indicate 5 mm. Scale bar in **f** indicates 2 mm. Scale bar in **g** indicates 1 mm and inset scale bar indicates 200 µm.

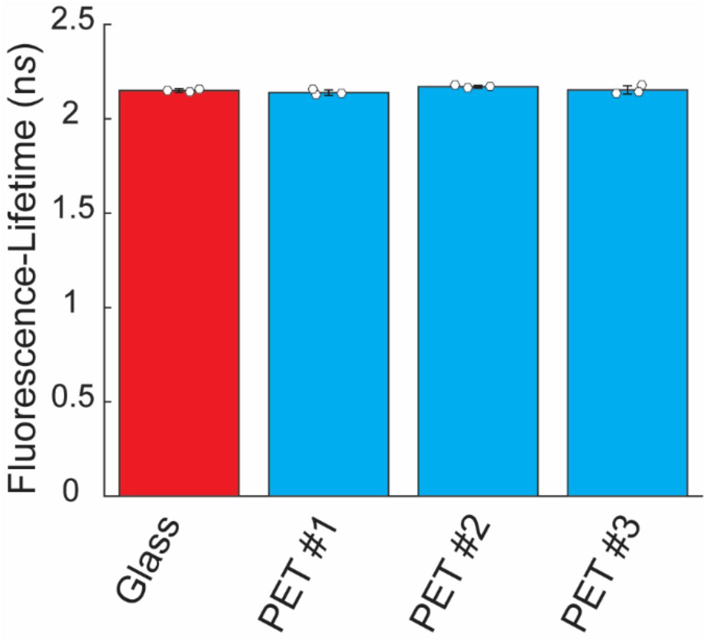

Supplementary Figure 4. Fluorescence-lifetime imaging microscopy (FLIM): Bar plots overlaid with the dot plots of fluorescence-lifetimes of YG beads measured while imaging through three samples of PET and glass coverslips (n = 3 measurements in each substrate). Error bars indicate s. d.

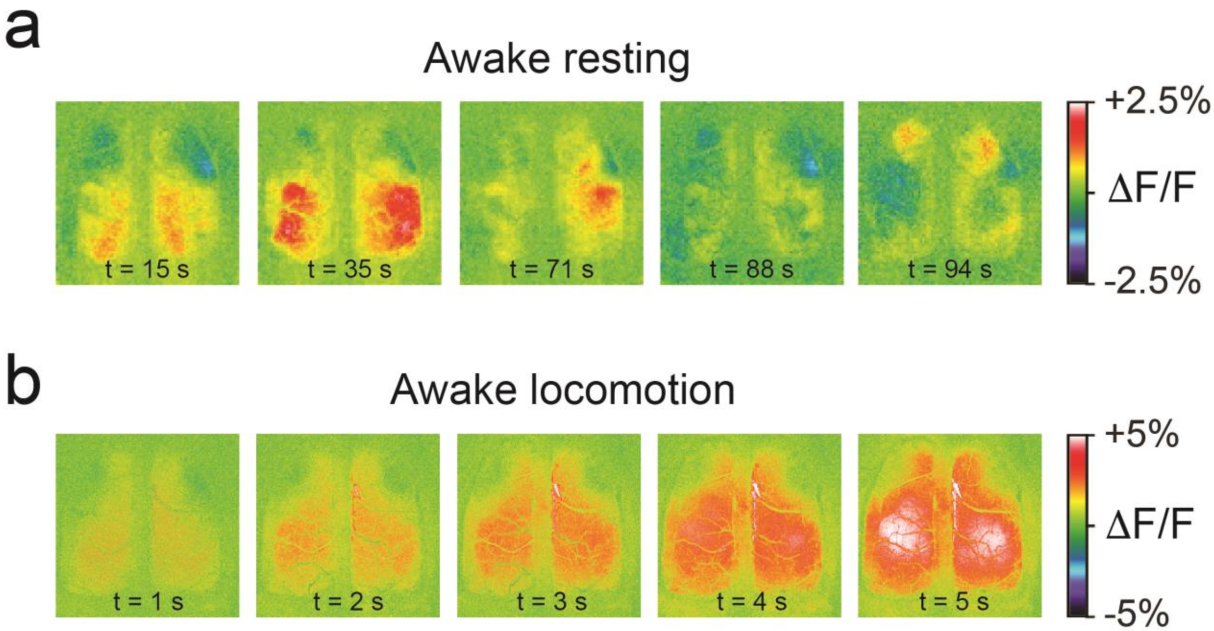

Supplementary Figure 5. Widely distributed cortical activity during awake behavior: (a) Mesoscale Ca^{2+} dynamics, visualized as changes in fluorescence ($\Delta F/F$) during a period of awake resting. **(b)** Mesoscale Ca^{2+} dynamics visualized as changes in fluorescence ($\Delta F/F$) during a period of locomotion.

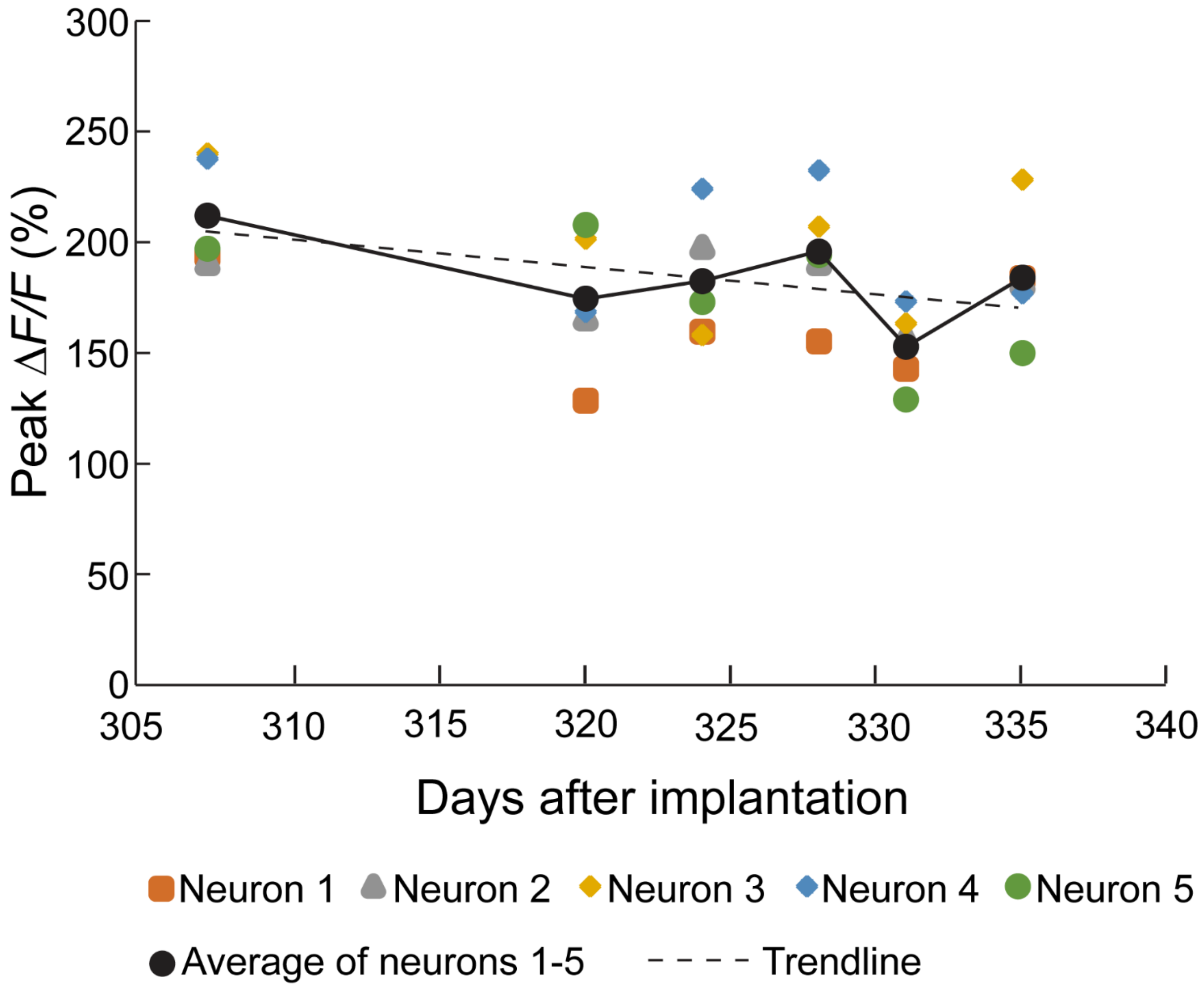

Supplementary Figure 6: Longitudinal Ca^{2+} imaging: Plot of peak *ΔF/F* of 5 neurons indicated in **Fig. 4e** over 6 imaging sessions. Dashed line indicates fitted trendline to the average data.

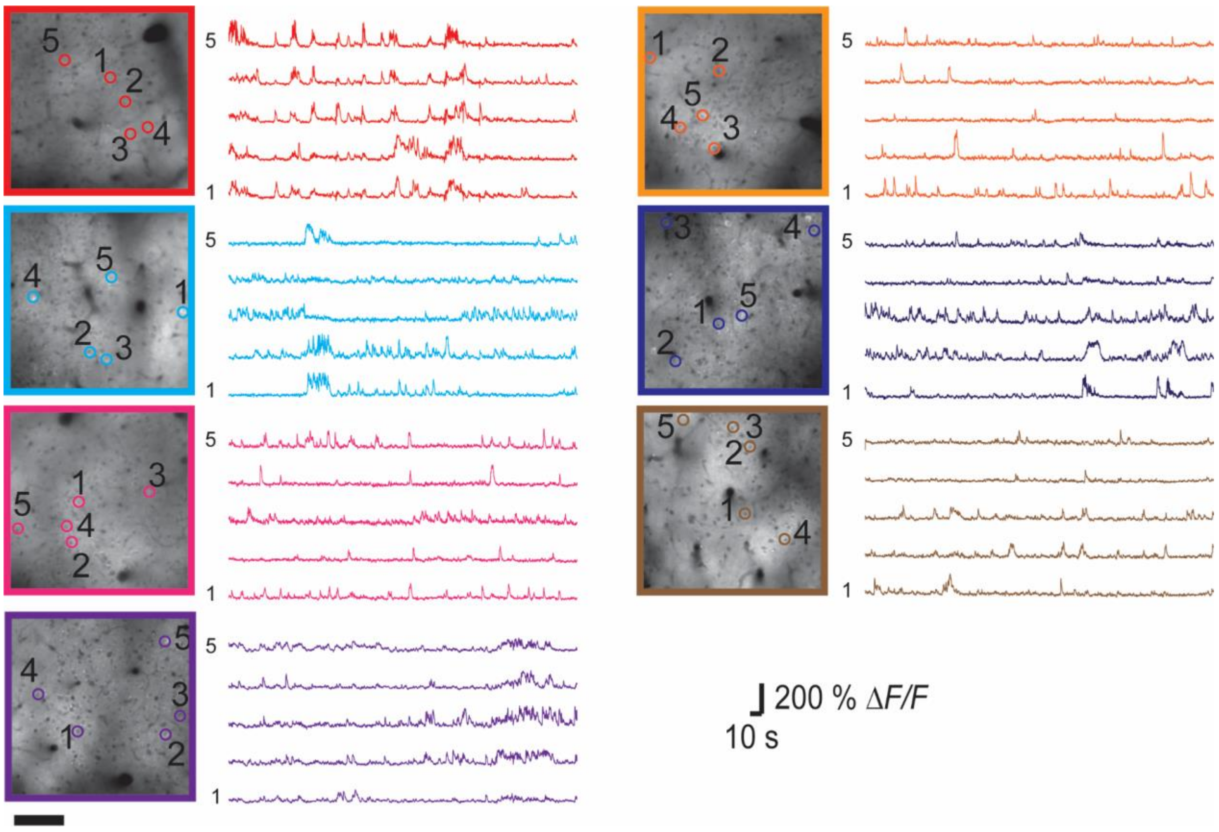

Supplementary Figure 7. Multisite 2P Ca^{2+} imaging during late stage implantation: Example 2P images from layer 2/3 neurons recorded at seven randomly accessed sites in a Thy1-GCaMP6f mouse, 48 weeks (335 days) after See-Shell implantation. Individual randomly selected neurons (1-5) are outlined by open circles to show Ca^{2+} transients. Scale bar indicates 100 μm.

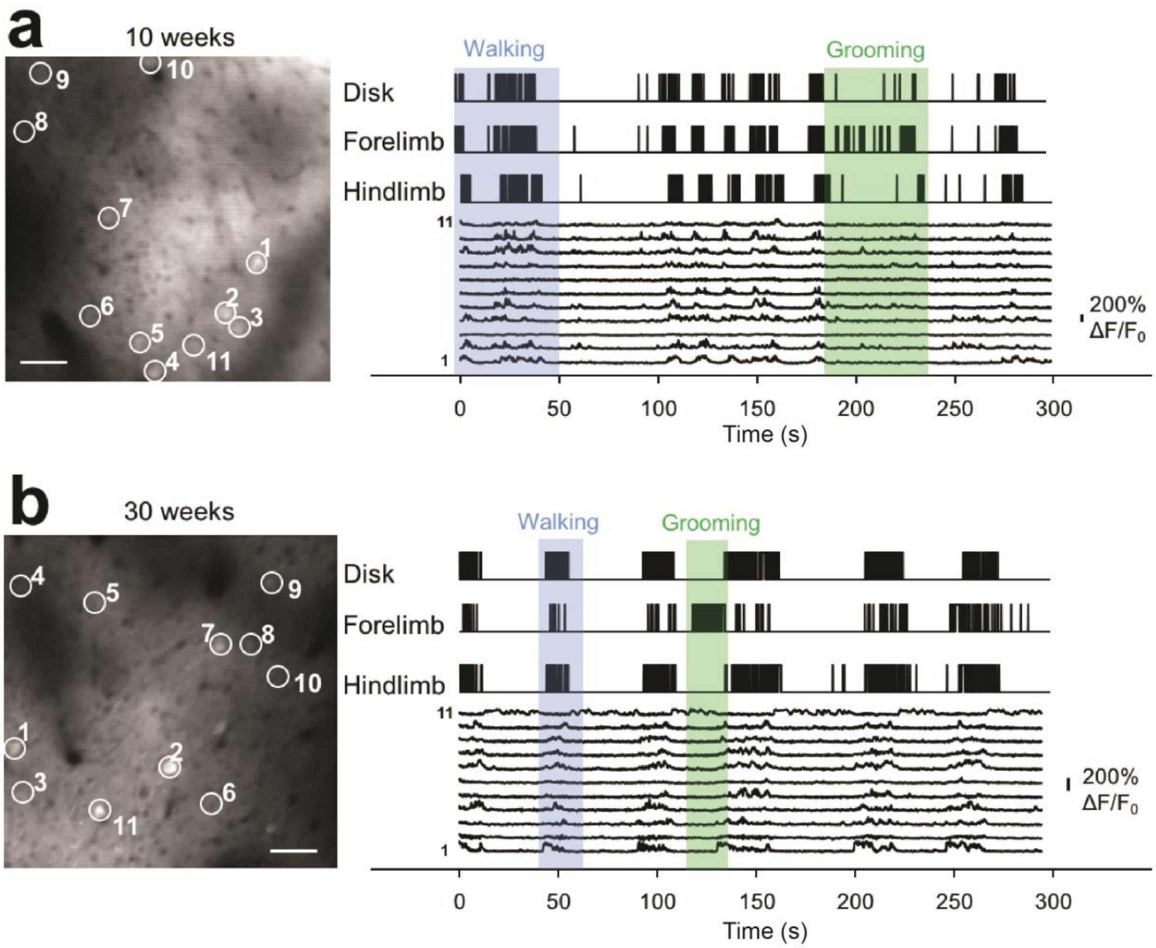

Supplementary Figure 8. Chronic 2P imaging and behavioral monitoring: (a) *Left:* Example 2P image from layer 2/3 in the primary motor cortex of a Thy1-GCaMP6f mouse 10 weeks after See-Shell implantation. Individual select neurons (1-11) are outlined by open circles, to show Ca^{2+} transients. Scale bar indicates 50 μm. *Right:* Monitoring changes in disk, forelimb, and hindlimb positions with high-speed cameras shows various behaviors such as walking (blue shaded area) and grooming (green shaded area). Single cell Ca^{2+} activity from the select neurons in the 2P image tend to show transients during periods of walking, but not grooming. **(b)** *Left:* Example 2P image from layer 2/3 in the primary motor cortex of the same Thy1-GCaMP6f mouse 30 weeks after See-Shell implantation. Optical clarity is still sufficient to see individual neurons (1-11). Scale bar indicates 50 μm. *Right:* Similar behaviors such as walking (blue shaded area) and grooming (green shaded area) can still be observed along with single cell Ca^{2+} activity (select neurons in 2P image) at 30 weeks as seen in imaging sessions at 10 weeks (**a**).

Supplementary Table 1. Light Transmission Measurement

**Wavelength (nm)**	Glass Bottom Dish	PET Sample #1	PET Sample #2	PET Sample #3
700	91.43	90.54	89.64	91.05
740	92.34	91.41	90.24	91.83
800	92.57	90.66	89.70	90.55
890	93.15	91.94	91.53	92.47
980	92.09	90.47	90.67	91.48
1040	94.16	93.19	92.94	93.19
1100	93.71	91.02	91.92	91.92
1200	92.86	90.11	90.11	91.21
**Mean**	**92.79**	**91.17**	**90.85**	**91.71**
**Standard Deviation**	**0.82**	**0.94**	**1.10**	**0.78**
