# Supplementary material for: Cortex-wide neural interfacing via transparent polymer skulls
Source: Nat Commun. 2019 Apr 2;10:1500. doi: 10.1038/s41467-019-09488-0 (PMC6445105; doi:10.1038/s41467-019-09488-0)
Supplement: Supplementary file 3 — Description of Additional Supplementary Files [file 41467_2019_9488_MOESM3_ESM.pdf]

Description of Additional Supplementary Files

File Name: Supplementary Movie 1
Description: Mesoscale imaging of spontaneous changes in fluorescent intensity in the dorsal cortex of a Thy1-GCaMP6f mouse during awake head-fixed behavior

File Name: Supplementary Movie 2
Description: 2P imaging of layer 2/3 neurons in the hindlimb region or the primary motor cortex of a Thy1-GCaMP6f mouse during awake head-fixation. Inset: pseudo-color images of change in fluorescent intensity in 350 µm x 350 µm field of view (FOV).

File Name: Supplementary Data 1
Description: File archive of CAD design files of See-Shell components.
