# Supplementary material for: Cortex-wide neural interfacing via transparent polymer skulls
Source: Nat Commun. 2019 Apr 2;10:1500. doi: 10.1038/s41467-019-09488-0 (PMC6445105; doi:10.1038/s41467-019-09488-0)
Supplement: Supplementary file 7 — Reporting Summary [file 41467_2019_9488_MOESM7_ESM.pdf]

natureresearch

Corresponding author(s): SUHASA KODANDARAMAIAH

Last updated by author(s): Feb 21, 2019

Reporting Summary

Nature Research wishes to improve the reproducibility of the work that we publish. This form provides structure for consistency and transparency in reporting. For further information on Nature Research policies, see Authors & Referees and the Editorial Policy Checklist.

Statistics

For all statistical analyses, confirm that the following items are present in the figure legend, table legend, main text, or Methods section.

n/a	Confirmed	
☐	☒	The exact sample size (*n*) for each experimental group/condition, given as a discrete number and unit of measurement
☐	☒	A statement on whether measurements were taken from distinct samples or whether the same sample was measured repeatedly
☐	☒	The statistical test(s) used AND whether they are one- or two-sided<br>*Only common tests should be described solely by name; describe more complex techniques in the Methods section.*
☒	☐	A description of all covariates tested
☒	☐	A description of any assumptions or corrections, such as tests of normality and adjustment for multiple comparisons
☐	☒	A full description of the statistical parameters including central tendency (e.g. means) or other basic estimates (e.g. regression coefficient) AND variation (e.g. standard deviation) or associated estimates of uncertainty (e.g. confidence intervals)
☐	☒	For null hypothesis testing, the test statistic (e.g. *F*, *t*, *r*) with confidence intervals, effect sizes, degrees of freedom and *P* value noted<br>*Give P values as exact values whenever suitable.*
☒	☐	For Bayesian analysis, information on the choice of priors and Markov chain Monte Carlo settings
☒	☐	For hierarchical and complex designs, identification of the appropriate level for tests and full reporting of outcomes
☒	☐	Estimates of effect sizes (e.g. Cohen's *d*, Pearson's *r*), indicating how they were calculated

Our web collection on statistics for biologists contains articles on many of the points above.

Software and code

Policy information about availability of computer code

Data collection	Multi SPC Software was used to collect time domain fluorescent lifetime images. Two-photon imaging data was acquired using Leica Application Suite software. Wide-field 1P imaging data was acquired using Metamorph. All electrophysiology data was collected using Intan Technologies Inc.'s open source RHD 2000 software.
Data analysis	SPCImage software (Becker & Hickl GbmH, Berlin, Germany) was used to analyze the fluorescence lifetime decay curves. All image analyses were performed in Fiji (ImageJ) and Matlab. Statistical analyses were performed in R and Matlab (The Mathworks Inc.) using standard scripts and functions. No custom code was used.

For manuscripts utilizing custom algorithms or software that are central to the research but not yet described in published literature, software must be made available to editors/reviewers. We strongly encourage code deposition in a community repository (e.g. GitHub). See the Nature Research guidelines for submitting code & software for further information.

Data

Policy information about availability of data

All manuscripts must include a data availability statement. This statement should provide the following information, where applicable:

- Accession codes, unique identifiers, or web links for publicly available datasets
- A list of figures that have associated raw data
- A description of any restrictions on data availability

We have now included a data availability statement, along with Source data file.

October 2018

Field-specific reporting

Please select the one below that is the best fit for your research. If you are not sure, read the appropriate sections before making your selection.

☒ Life sciences ☐ Behavioural & social sciences ☐ Ecological, evolutionary & environmental sciences

For a reference copy of the document with all sections, see nature.com/documents/nr-reporting-summary-flat.pdf

Life sciences study design

All studies must disclose on these points even when the disclosure is negative.

Sample size	We did not compute the sample size, because the experiments were performed to demonstrate the efficacy of the methodology presented, and not to make statistical claims about a biological phenomena or mechanism.
Data exclusions	No data was excluded in the analyses.
Replication	In this study the main biological units are mice. All experiments were performed on multiple mice (n = 3 or greater) and were reproducible. Some GCaMP6f implanted mice were used in multiple experiments -for e.g., to perform 2P imaging as well as mesoscale imaging during cortical microstimulation.
Randomization	We did not perform any randomization, because the experiments were performed to demonstrate the efficacy of the methodology presented, and not to make statistical claims about a biological phenomena or mechanism.
Blinding	Blinding was used for analyses of histology data. All other experiments were conducted to characterize performance of the polymer skulls across all conditions without specific hypotheses about whether the implants performed better in certain conditions. Thus we did not find blinding necessary.

Reporting for specific materials, systems and methods

We require information from authors about some types of materials, experimental systems and methods used in many studies. Here, indicate whether each material, system or method listed is relevant to your study. If you are not sure if a list item applies to your research, read the appropriate section before selecting a response.

Materials & experimental systems

n/a	Involved in the study	
☐	☒	Antibodies
☒	☐	Eukaryotic cell lines
☒	☐	Palaeontology
☐	☒	Animals and other organisms
☒	☐	Human research participants
☒	☐	Clinical data

Methods

n/a	Involved in the study	
☒	☐	ChIP-seq
☒	☐	Flow cytometry
☒	☐	MRI-based neuroimaging

Antibodies

Antibodies used	Primary antibody-Monoclonal Anti-Glial Fibrillary Acidic Protein antibody produced in mouse (G3893-.2ML, Sigma Aldrich)<br>Secondary antibody conjugated with fluorophores (anti-mouse Alexa 488, ab150117, abcam)
Validation	Sigma-Aldrich warrants, that at the time of the quality release or subsequent retest date this product conformed to the information contained in this publication.

Animals and other organisms

Policy information about studies involving animals; ARRIVE guidelines recommended for reporting animal research

Laboratory animals	We used both male and female mice form C57BL/6, Thy1-YFP (#003709, Jackson Laboratories), Thy1-GFPm (#007788, Jackson Laboratories) and Thy1-GCaMP6f mice (#024276, Jackson Laboratories) strains- age : 8 to 44 weeks
Wild animals	The study did not involve wild animals.
Field-collected samples	The study did not involve samples collected from the field.
Ethics oversight	All of the animal studies were approved by and conducted in conformity with the Institutional Animal Care and Use Committee of the University of Minnesota.

Note that full information on the approval of the study protocol must also be provided in the manuscript.
